# Supplementary material for: MRI Characteristics Accurately Predict Biochemical Recurrence after Radical Prostatectomy
Source: J Clin Med. 2020 Nov 26;9(12):3841. doi: 10.3390/jcm9123841 (PMC7760513; doi:10.3390/jcm9123841)
Supplement: Supplementary file 1 [file jcm-09-03841-s001.pdf]

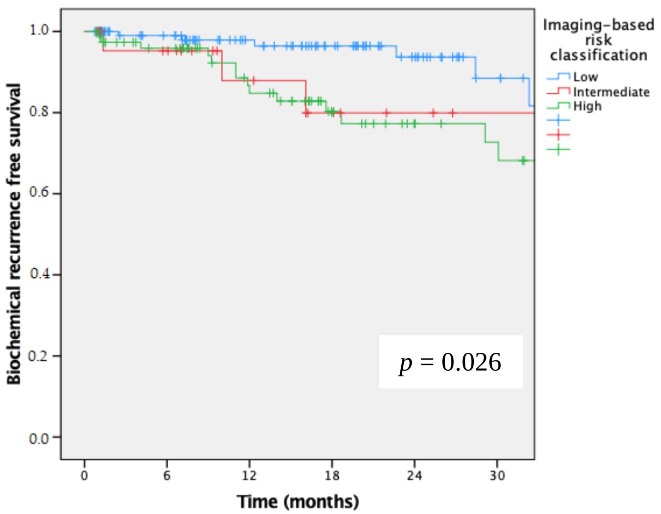

Number at risk						
Low	*120*	*94*	*67*	*50*	*31*	*16*
Intermediate	*24*	*19*	*12*	*8*	*5*	*3*
High	*78*	*64*	*45*	*28*	*19*	*16*

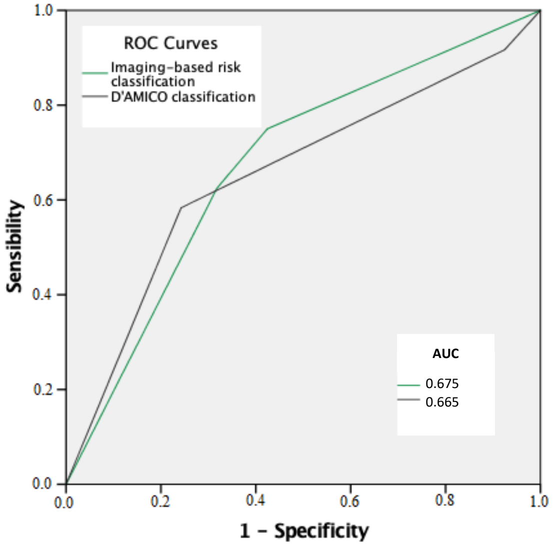

(a) Center 1

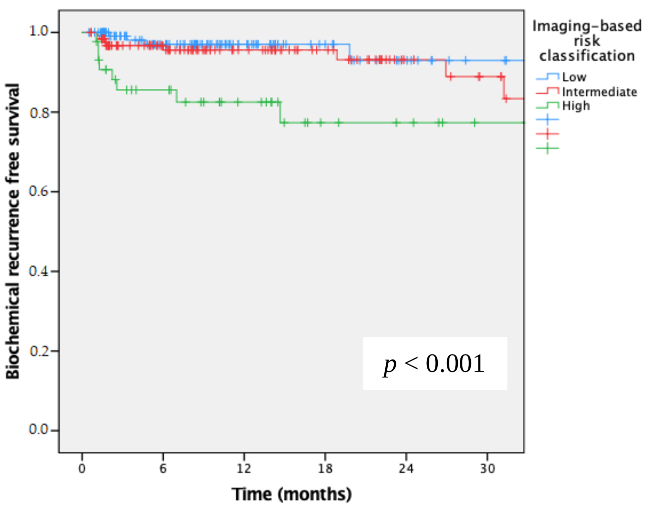

Number at ri						
Low	*128*	*85*	*45*	*27*	*16*	*9*
Intermediate	*127*	*88*	*58*	*41*	*23*	*18*
High	*44*	*30*	*21*	*11*	*9*	*5*

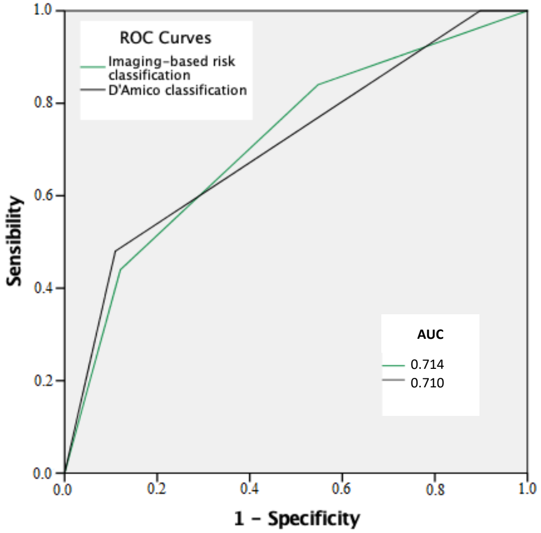

(b) Center 2

Supplementary Figure 1. Imaging-based risk classification in each Center. Above recurrence free survival curve (blue: low risk, red: intermediate risk, green: high risk).
